# Pooled coverage of community based health insurance scheme enrolment in Ethiopia, systematic review and meta-analysis, 2016–2020

**DOI:** 10.1186/s13561-022-00386-8

**Published:** 2022-07-12

**Authors:** Ahmed Tahir, Abdulahi Omer Abdilahi, Abdifatah Elmi Farah

**Affiliations:** Public Health Department, College of Medicine and Health Science, Jigjiga University, P.O.Box: 1020, Jijiga, Ethiopia

**Keywords:** CBHI, Coverage, Systematic review, Ethiopia

## Abstract

**Background:**

Community Based Health Insurance (CBHI) is a type of health insurance program that provides financial protection against the cost of illness and improving access to health care services for communities engaged in the informal sector. In Ethiopia, the coverage of CBHI enrolment varies across regions and decision of household enrolment is affected by different factors. There are pocket studies on CBHI scheme with different coverage in Ethiopia and there is no pooled study on CBHI enrolment coverage in Ethiopia for better understanding the scheme and decision making. The aim of this systematic review and meta-analysis was to identify the pooled coverage of CBHI enrolment in Ethiopia to understand its policy implications.

**Methods:**

The systematic review and meta-analysis was done by adhering the PRISMA guideline with exhaustive search in PubMed/Medline, HINARI, SCOPUS and Google scholar complemented by manual search. Two authors independently selected studies, extracted data, and assessed quality of studies. The I^2^ test statistic was used to test heterogeneity among studies. The overall coverage of CBHI scheme was estimated by using random-effects model.

**Result:**

Among 269 identified, 17 studies were included in this meta-analysis and the overall coverage of CBHI scheme was 45% (95% CI 35%, 55%) in Ethiopia. The sub-group analysis shows higher enrolment rate 55.97 (95%CI: 41.68, 69.77) in earlier (2016–2017) studies than recent 37.33 (95%CI: 24.82, 50.77) studies (2018–2020).

**Conclusion:**

The pooled coverage of CBHI enrolment is low in Ethiopia compared the national target of 80% set for 2020. It is also concentrated in only major regions of the country. The finding of the study helps national decision making for CBHI scheme service improvement. Due attention to be given to improving geographic expansion of CBHI and to the declining coverages with in the CBHI implementing regions by addressing the main bottlenecks restraining coverages.

**Trial registration:**

The protocol of this systematic review and meta-analysis was published in PROSPERO with registration number: CRD42021252762.

**Supplementary Information:**

The online version contains supplementary material available at 10.1186/s13561-022-00386-8.

## Background

Community Based Health Insurance (CBHI) is a type of health insurance program that provides financial protection against the cost of illness and improving access to health care services for communities engaged in the informal sector. There are two types of Health insurance: Social Health Insurance (SHI) for the formal sector and CBHI for the rural & urban informal sector.

Universal health coverage means that all people have access to health services they need when and where they need them without financial hardship [1]. It includes the full range of essential health services, from health promotion to prevention, treatment, rehabilitation, and palliative care [1, 2]. The progress towards this aspiration seems poor [3] particularly for countries whose fiscal capacity is low and whose social health insurance for the employed sector is absent or very small, thus limiting the mobilization of additional resources from payroll contributions.

Financing health care in most developing countries greatly relies on out-of-pocket payments which contributes to unacceptably high burdens of preventable diseases and deaths [4] with most donors and global health initiatives such as the Global Fund focusing on specific diseases or interventions rather than the broader health system.

Countries with a high share of out-of-pocket payments are more likely to have a high proportion of households facing catastrophic health expenditure—defined as spending more than 40% of household consumption expenditure, excluding food, on health, more than 25% of non-food consumption expenditure of households on health, or more than 10% of total household consumption expenditure on health [5].

Many lower and middle-income countries (LMICs) are getting healthcare service with equity issues to its citizens as health services lacks funds to finance [6, 7]. Health care expenditure is low (12%) in low income countries which increased the burden of out-of-pocket payment (OPP) [8, 9]. This is one of the obstacles to accessing healthcare in Ethiopia too [10]. Thus this made reasonable to advocate community-based health insurance program (CBHI) to be able financing healthcare [3]. However, low household enrolment rate challenged the accessibility of healthcare [7, 8].

The available evidence clearly demonstrates that health insurance can be an alternative to user fees as a health financing mechanism. The strong evidence that Community Based Health Insurance( CBHI) and Social Health Insurance (SHI) can improve financial protection and enhance service utilization patterns [11], but the weaker evidence that CBHI and SHI can foster social inclusion of specific vulnerable groups in Low middle income countries (LMIC) [12, 13].

Ethiopia endorsed a health care financing strategy in 1998 that envisioned a wide range of reform initiatives launch of health to remove financial barriers, enhance equity, increase health service utilization rate and improve quality of care by increasing resources available for health facilities [14]. In its revised strategy (2017–2025), Ethiopia envisioned to ensure universal health coverage through primary health care by 2035 [15]. The Per capita spending is $33.2 which is far below the globally recommended $86 per capita estimated to make essential health care services available in low-income countries. In terms of spending, the government spends 8% of government budget on health sector which is below the Abuja target of 15% and the out-of-pocket expenditure is also still high standing at 31% of total health expenditure [16].

In Ethiopia, CBHI scheme was launched and piloted in 2011 and is among the avenues designed contribute to reduction of out-pocket payments and realization of universal health coverage. The contribution (premium) is collected from the households at the pre-set flat rate meaning equal amount of payment is levied to every household regardless of their household characteristics such income and family size [17].

The national CBHI enrolment coverage was 50% in 2020, while the 2020 target was to achieve with 80% coverage. The majority of these CBHI woreda are located in four developed regions and the capital city of the country [18]. The coverage is also varies across regions and decision of household enrolment affected by different factors [19–22], with the existing regional variation; only 28% of the communities were enrolled to the program, as reported in the Ethiopian demographic and health survey (EDHS, 2019) [23]. Moreover, membership dropout has been also another challenge for low enrolment in Ethiopia [7, 24]. The target of Ethiopia’s HSTP1(Health Sector Transformation Plan 1) was that 80% of all districts achieve 80% coverage of CBHI in 2020, close to 825 districts out of the estimated 1100 districts in the country are implementing the scheme which translates to 75% geographic coverage nationally.

There are pocket studies on CBHI scheme with different service coverage in Ethiopia and there is no pooled study on CBHI enrolment coverage in Ethiopia for better understanding the scheme and decision making. The aim of the manuscript is to present a systematic review and meta-analysis in order to identify the pooled coverage of CBHI enrolment for better picture and decision making in Ethiopia and to understand its policy implications.

## Methods

### Search strategy

The Preferred Reporting Items for Systematic review and Meta-Analysis (PRISMA) guideline was used to conduct this systematic review and meta-analysis [25]. An exhaustive and comprehensive search was performed to recognise all relevant studies available in English regardless of their publication status (published, unpublished, in progress or in press). The data was searched from the following electronic databases: PubMed/Medline, HINARI, SCOPUS and Google scholar. In addition to this, other manual search on academic in local universities including Addis-Ababa University was also done to include important un-published papers. The following search strategy was used to find published articles on CHBI in Ethiopia:


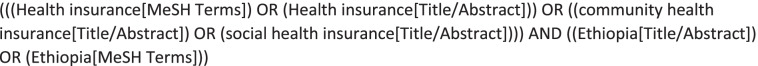


The protocol of this study was published on PROSPERO website with the following registration number: CRD42021252762.

### Inclusion and exclusion criteria

We included observational quantitative studies that reported coverage of CBHI enrolment in Ethiopia. For this review. To be included, the studies has to report the following primary outcome: Coverage of CHBI scheme enrolment (informal sector) and cross-sectional study design. Studies reporting social health and private health insurance (formal sector) were excluded.

### Study selection

Two authors (Ahmed T. and Abdulahi O.) independently used the inclusion criteria for identification of titles and abstracts of studies and selected papers full texts were reviewed using the eligibility criteria. Any paper matches excluded criteria was simply excluded.

### Quality assessment

To assess the quality of studies included, a tool containing 9 criteria was used and adapted from Hoy 2012 [26]. The following variables were assessment criteria: (1) representation of the population, (2) sampling frame, (3) methods of participants’ selection, (4) non-response bias, (5) data collection directly from subjects, (6) acceptability of case definition, (7) reliability and validity of study tool, (8) mode of data collection, (9) appropriateness of numerator and denominator.

The total score was used as the summary assessment for the risk of bias: low risk bias (0–3), moderate risk bias (4–6) and high risk bias (7–9). Each study was evaluated by two authors (Ahmed T. and Abdulahi O.). In case of any gap seen during assessment of a study between the authors, a final decision was taken by consensus. In summary, all assessed studies (moderate and poor quality) were included in this review to reduce publication bias (supplementary file 1, Table 1).

**Table 1:** Characteristics of included studies of meta-analysis of CBHI scheme coverage, Ethiopia, 2016–2020

Studies	Survey year	CBHI enrolment	Total population	Residence	Sampling procedure	Risk of bias	Setting
Abdilwohab MG	2018	273	820	SNNP/Arba Minch Town	Systematic random sampling	Low risk bias	Urban
Ashagrie B	2020	63	584	Amhara/Dera District	systematic random sampling	Low risk bias	Mixed (82% Rural)
Nageso D	2020	81	646	SNNP/Boricha district, Sidama Zone	simple random sampling	Low risk bias	Mixed(95% Rural)
Gebru T	2020	982	1856	SNNP/Dale Woreda (Yirgalem)	simple random sampling	Low risk bias	Urban
Mirach TH	2017	401	690	Amhara/ West Gojjam zone	multistage sampling	Low risk bias	Mixed
Simieneh MM	2016	205	410	Amhara/Aneded district	multistage sampling	Low risk bias	Rural
Mekonen AM	2018	224	455	Amhara/Tehuledere district	Multi stage sampling	Low risk bias	Rural
Workneh SG	2017	471	511	Amhara/Tehuledere district	A systematic random sampling	Low risk bias	mixed (92 rural)
Atnafu A	2017	111	226	Amhara/North-west	simple random sampling	Low risk bias	mixed
Atafu A,	2017	832	2008	Amhara/North-west	multi‐stage sampling	Low risk bias	mixed(91% rural)
Tilahun H	2016	326	652	Amhara/ Achefer woreda	mutistage sampling	Moderate risk bias	Rural
Dera G	2020	222	382	Oromia/Liban district	Systematic random sampling	Low risk bias	Rural
Negash K	2020	148	584	Oromia/Gumbichu Woreda	Systematic random sampling	Low risk bias	Rural
Shiferaw S	2017	272	630	Oromia/Sabata Hawas Woreda	simple random sampling	Low risk bias	Urban
derseh da	2019	349	634	Benishangul /Bambasi Woreda	simple random sampling	Low risk bias	Urban
Zerihun B	2020	82	405	SNNP/Amaro District	simple random sampling	Low risk bias	Mixed
Eseta WA	2020	432	634	Oromia/Manna District, Jimma Zone	multistage sampling	Low risk bias	Mixed (88% Rural)
Total			12,127				

### Data extraction

The two authors (AT & AO) independently extracted data for each study using prepared an excel sheet form. For each identified study, survey year, CBHI enrolment rate, sample size, residence by region, sampling procedure and setting of the study were extracted for analysis.

### Data analysis and synthesis

Stata (version 13; Stata Corp, College Station, TX) software was used to estimate the pooled prevalence of CBHI enrolment coverage. The heterogeneity was detected if squared I (I^2^) statistic would be significant at least 50% [27]. To analyse heterogeneity, we did sensitivity analysis by selecting studies with small sample size and old (conducted earlier time) ones. In addition, subgroup analysis by survey year, regional states and study settings (rural, urban and mixed), and reported the remaining heterogeneity as it is. We used random effect model with 95% CI to estimate the pooled prevalence of CBHI enrolment. The study used forest plot and tables to show narrative synthesis for details. Lastly, Asymmetry funnel plots and *P* < 0.05 in Egger weighted regression and Begg rank correlation test were considered to assess publication bias [28].

## Result

**Fig. 1:**
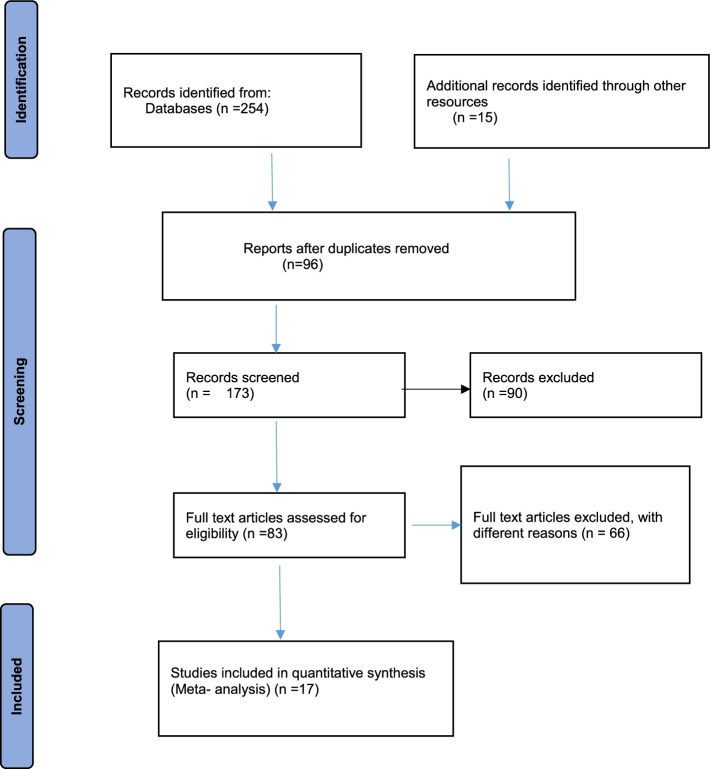
PRISMA flow diagram indicating selection of studies for systematic review and meta-analysis on CBHI enrolmen coverage in Ethiopia

A total of 269 studies were captured by searching through different literature database and other searches, of which 96 studies were removed due to duplication and 90 were not in line with the aim of the study and therefore excluded by title and abstract review. Furthermore, 83 full text publications were evaluated for eligibility, with 66 being rejected due to the following details: 27 of the articles’ outcomes differed from the intended study outcome, design; case control (*n* = 5) and geographical analysis (*n* = 2), as well as social health insurance (*n* = 15). Finally, only 17 studies were included for this meta-analysis (Fig. 1).

### Description of included studies

A total of 17 studies were included for this systematic review and meta- analysis. All included studies were cross sectional by design and conducted in only four regions of Ethiopia. Included studies done from 2016 to 2020 which composed 12,127 households were used to estimate CBHI enrolment coverage in Ethiopia. The majority (41.2%) of these studies were conducted in Amhara region [29–36], followed by Oromia [37–40] and SNNP [19, 41–43] and then Benshangul Gumuz [44]. Moreover, majority [19, 29, 30, 33–35, 40, 43] of the studies were conducted in mixed setting with significant proportion of rural. Whilst, four studies [39, 41, 42, 44] were conducted in purely urban setting the rest five studies [31, 32, 36–38] in rural setting (Table 1).

### Risk of bias

Risk of bias tool of prevalent study was used to assess quality of each and every included the study and tested through nine different criteria (27). The two authors (AT &AO) were thoroughly assessed each included study one by one by using questions and procures discussed at above subtopic of quality assessment. The majority (94%) of 17 included studies were low risk of bias [19, 29–35, 37–44] while only one study was moderate risk of bias (40) according to our summary assessment finding (Table 1).

### Publication bias

**Fig. 2:**
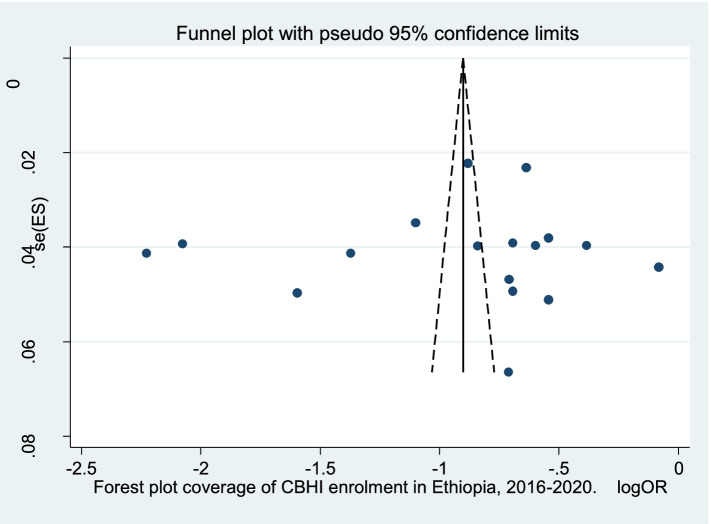
Forest plot coverage of CBHI enrolment in Ethiopia, 2016–2020

The small study effect of the included studies was visually and statistically analysed. However, included studies were symmetry on the funnel plot that shows no any evidence of publication bias (Fig. 2). Furthermore, Egger’s test revealed that there was no publication bias (*p*-value = 0.997).

### Heterogeneity

Heterogeneity was assessed using I^2^ statistics test. A value of 50% and above of I^2^ and P-value of less than 0.05 was used to detect presence of high heterogeneity. In this study, the I^2^ test result shows high (99.17%, *P*-value < 0.01) heterogeneity for the study. Therefore, we used random effect model to estimate pooled coverage for CBHI enrolment in Ethiopia. Furthermore, subgroup and sensitivity analysis were also conducted for better understanding.

### Subgroup analysis

Since there was statistically substantial heterogeneity, I-square test statistics less than 0.05 (99.17%, *P*-value < 0.01), a subgroup analysis was performed. The goal of the analysis was to pinpoint the source of heterogeneity so that the results could be properly interpreted. We performed a subgroup meta-analysis of the included studies by region, survey year and setting of the study. However, no significant variable was revealed in the subgroup analysis to explain the heterogeneity in this review. As a result, other factors not considered in this study can explain the heterogeneity (Table 2).

**Table 2:** Subgroup analysis coverage of CBHI enrolment by region, date of the survey, and setting of the study using I^2^ test for heterogeneity in Ethiopia, 2016- 2020

Characteristics	Coverage (%)	95% CI	*P*-Value	I^2^ (%)	Weight (%)
Region of the study					
Amhara	50.36	33.8, 66.89	0.00	99.33	47.01
Oromia	48.51	29.64, 67.6	0.00	98.85	23.52
SNNP	28.68	11.78, 49.45	0.00	99.36	23.58
Benishangul-Gumuz	55.05	51.16, 58.88	0.00	0	5.89
Date of survey					
2016–2017	55.97	41.68, 69.77	0.00	98.99	41.14
2018–2020	37.33	24.82, 50.77	0.00	99.22	58.86
Setting of the study					
Urban	46	36, 56	0.00	97.27	23.61
Rural	46	35, 58	0.00	97.16	29.37
Mixed	44	24, 64	0.00	99.58	47.03

### Sensitivity analysis

Sensitivity analysis was done to determine the impact of each study on heterogeneity by removing studies with small sample sizes (*n* < 100) and relatively old (conducted earlier) one by one at a time. However, the removed studies had nothing to do with the heterogeneity of estimates as the result remains the same (Table 3).

**Table 3:** Sensitivity analysis to estimate pooled CBHI enrolment coverage in Ethiopia, 2016–2020

Serial No	Study omitted	Reasons of Omission	Pooled coverage of CBHI enrolment(95% CI)	I^2^ Values
1	Ashagrie B., 2020	Small sample size(63)	47.42(38.12, 56.81)	99.03
2	Nageso D., 2020	Small sample size(81)	47.26(37.90, 56,71)	99.03
3	Zerihun B., 2020	Small sample size(82)	46.59(36.64, 56. 68)	99.17
4	Simieneh MM., 2016	Date is too early(2016)	44.62(34.44, 55.03)	99.22
5	Tilahun H., 2016	Date is too early(2016)	44.62(34.33, 55.14)	99.22

### Coverage of CBHI enrolment

**Fig. 3:**
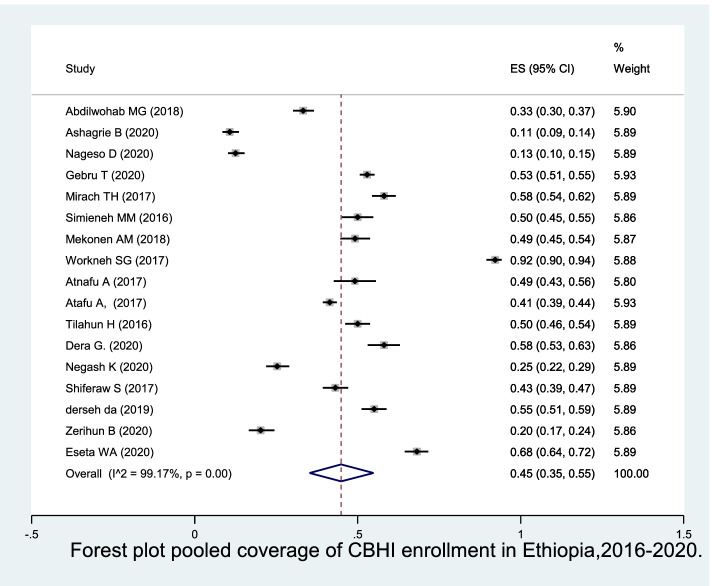
Forest plot pooled coverage of CBHI enrolment in Ethiopia, 2016–2020

The pooled CBHI enrolment coverage was 45% (95% CI 35%, 55%) in Ethiopia based on 17 studies and random effect model estimate (Fig. 3).

It varies from 10.8% of 584 households in Dera District of Amhara region to 92.2% of 511 households in Tehuledere district of Amhara region.

Subgroup analysis of CBHI enrolment coverage was done by region, date of survey, and setting of the study based on 17 studies. The coverage of CBHI enrolment was 55.05% (95%CI: 51.16, 58.88) in Benishangul-Gumuz region, followed by Amhara 50.36% (95%CI: 33.8, 66.89), Oromia 48.51%( 95%CI: Oromia and SNNP 28.68%( 95%CI: 11.78, 49.45) regions respectively. However, the lowest weight comes from Benishangul-Gumuz region while the highest weight come from Amhara region.

According to date of survey, the 17 studies were categorized into studies conducted from 2016–2017(41.2%) and studies conducted from 2018 to 2020(58.8%). The coverage of CBHI enrolment was higher among studies conducted earlier time (55.97%, 95%CI: 41.68, 69.77) compared to resent studies [37.33%, 95%CI: 24.82, 50.77].

Moreover, regarding study setting, 17 studies were divided into rural (29.4%), urban (23.5%) and mixing (47.1%) settings. The coverage of CBHI enrolment was relatively lower (44%, 95%CI: 24, 64) in studies done in mixed setting compared to urban (46%, 95%CI: 36, 56) and rural (46%, 95%CI: 35, 58) settings (Table 2).

## Discussion

This systematic review and meta-analysis study aimed at estimating the pooled CBHI enrolment coverage in Ethiopia based on the available studies. Subgroup analysis of CBHI enrolment coverage was done by region, date of survey, and setting of the study based on 17 studies.

The pooled CBHI coverage was found to be 45% (95% CI 35%, 55%), the lowest coverage (10.8% of 584 households) was reported from Dera district of Amhara region in 2020, whilst the highest coverage (92.2% of 511 households) was reported from Tehuledere district of Amhara region in 2017.

This pooled coverage is similar to findings from a study in Ethiopia indicating a coverage of 45.5% [45], but is far below the national target set for 2020 to achieve 80% coverage in 80% of districts throughout the country [18]. This below national target coverage is because the scheme is challenged by annual dropouts mainly ascribed to the voluntary nature of the scheme. It is also lower than a national coverage of 50% reported by Ethiopian Insurance Agency in 2020 [18] which might have a drawback of overestimation as it is a routine administrative report.

On the other hand, our reported coverage is higher than the coverage (28%) reported by mini-EDHS, 2019 [23] EDHS is a population-based survey covering all regions of Ethiopia given that significant number of regions did not initiate the scheme which dragged the national coverage down. A study in Ghana also reported a lower rate of (38%)enrolment than our reported coverage [46] in addition to other sub-Saharan African studies which have challenges of low enrolment rates [47, 48] except Rwanda which has achieved a higher coverage [49].

Despite national initiatives to address financial barriers (such as community-based health insurance, or CBHI with premium subsidy for indigents), socioeconomic disparities in health service utilization are very wide, even for services that do not require user fees, including reproductive maternal neonatal and child health (RMNCH) services [50]. The geographic disparity in the scale up of the scheme has a contribution too with CBHI much concentrated to the central regions of Oromia, Amhara, SNNPR than the peripheral regions witnessing recent initiations in addition to available evidence [51]. This systematic review was estimated from studies done mainly in four developed regions of Ethiopia: Amhara, Oromia, SNNP and Benishangul-Gumuz whilst the national coverage was based on similar regions as above including Tigray region and Addis-Ababa city in addition.

In sub-group analysis shows higher enrolment rate 55.97(95%CI: 41.68, 69.77) in earlier (2016–2017) studies than recent 37.33(95%CI: 24.82, 50.77) studies (2018–2020). This means CBHI scheme is not progressing towards its target (80%). The possible explanation of the decreasing coverage is high scheme dropout and low uptake in Ethiopia which may be due to affordability, quality of care and awareness issues of the scheme [8, 29, 40, 52, 53, 50]. Membership dropout is still a challenge to sustain CBHI program, and it is mainly due to the voluntary nature of the community bases health insurance system, in which wealthy and healthy people freely opt to join/not to join and or to exit from CBHI membership which erodes the core principle of solidarity [18, 54]. This is reaffirmed by a study in Uganda where 25% of households that had ever enrolled in insurance reported drop out in Uganda and other middle income countries [55, 56]. This review unlike the EDHS, 2019 finding, shows no difference between pure urban (46%) and rural settings in our study even though high proportions are rural in mixed setting.

Moreover, a different enrolment coverages were reported from regions of Ethiopia with highest coverage in Amhara region next to Benishangul-Gumuz region. However, the highest weight (47.01%) belongs to Amhara region and the lowest (5.89%) to Benishangul-Gumuz region in sub-group analysis. in a nutshell, none of the regions’ coverage is on track for national plan of 80% coverage.

### Strengths and limitations

Exhaustive searching on published and unpublished papers was done through manual and electronic strategy. The quality assessment was done by two independent authors and majority of included studies were low risk bias while only one study was moderate risk bias. Nevertheless high heterogeneity was observed (large I^2^) among included studies. The high heterogeneity might be due to different regions of Ethiopia where studies were conducted. However, the pooled CBHI coverage was estimated by using random –effects model and sub-group and sensitivity analysis to manage high heterogeneity. Some of the studies included in this review had small sample sizes, which may have influenced the findings of the pooled estimate. In addition, the studies in this meta-analysis were from north, south and west of the country where east part were not represented due to the unavailability of studies.

Since the scheme is not scaled up to some peripheral regions, the pooled coverage estimation doesn’t reflect the status of the mentioned regions.

## Conclusions

The pooled coverage of CBHI service enrolment is low in Ethiopia compared the national target of 80% set for 2020. It is also concentrated in only major regions of the country. The study finding helps for decision making as it is more refined (pooled data) than pocket study. Due attention to be given to improving geographic expansion of CBHI and to the declining coverages with in the CBHI implementing regions by addressing the main bottlenecks restraining coverage. Nationwide longitudinal study should be conducted to identify the challenges of CBHI enrolments coverage.

## Supplementary Information

**Additional file 1:** **Supplementary Table 1.** Quality assessment for risk of bias among included studies on CBHI coverage, Ethiopia, 2016-2020.

## Data Availability

It is not applicable for systematic review and meta-analysis.

## Abbreviations

CBHI: Community Based Health Insurance
SHI: Social Health Insurance
LMICs: Lower and Middle Income Countries
OOP: Out of Pocket
EDHS: Ethiopian Demographic and Health survey
HSTP: Health Sector Transformation Plan
